# Use of artificial primary teeth for endodontic laboratory research: experiments related to canal length determination

**DOI:** 10.1186/s12903-017-0420-3

**Published:** 2017-11-17

**Authors:** Anna Carolina V. Mello-Moura, Carmela R. Bresolin, Cacio Moura-Netto, André Ito, Angela T. Araki, José Carlos P. Imparato, Fausto M. Mendes

**Affiliations:** 1 Department of Pediatric Dentistry, School of Dentistry, University of São Paulo, São Paulo, Brazil; 2 School of Dentistry, Universidade Ibirapuera, São Paulo, Brazil; 3 School of Dentistry, Universidade Cruzeiro do Sul, São Paulo, Brazil; 4 São Leopoldo Mandic Institute and Research Center, Campinas, Brazil; 5 Faculdade de Odontologia da Universidade de São Paulo, Av. Lineu Prestes, 2227, São Paulo, SP 05508-000 Brazil

**Keywords:** Artificial teeth, Canal length determination, Endodontics, Primary teeth

## Abstract

**Background:**

Due to the scarcity of exfoliated/extracted human primary teeth with complete roots, artificial teeth were developed as an alternative to be used for educational and laboratory research purposes. This study aimed to assess the feasibility of using artificial primary teeth for conducting laboratory research through an experiment related to canal length determination, comparing artificial teeth with natural teeth.

**Methods:**

Thirty anterior and 21 posterior artificial teeth, and the same number of natural primary teeth were selected. After preparing the access cavity, the root canal length was determined by two examiners twice using three different methods: radiography and two electronic apex locators. Then, the actual root canal length was measured by inserting a K-file up to the apical foramen (reference standard). Accuracy was calculated using Bland-Altman analysis and intraclass correlation coefficient (ICC). The inter- and intra-examiner reproducibility was also calculated using the ICC.

**Results:**

The methods using the electronic apex locators showed better accuracy in both artificial and natural teeth. Trends observed with artificial primary teeth were similar to those observed with natural teeth, except for the results in artificial anterior teeth.

**Conclusions:**

The model of artificial teeth might be a good alternative for educational purposes; however, improvements are necessary to employ these teeth for research purposes when considering experiments for canal length determination.

**Electronic supplementary material:**

The online version of this article (10.1186/s12903-017-0420-3) contains supplementary material, which is available to authorized users.

## Background

The maintenance of primary teeth until the period of exfoliation is a major goal of pediatric dentistry, since they have a fundamental role in aesthetics, phonetics, function and in guiding permanent succedaneous teeth [1]. In some situations, such as trauma and extensive carious lesions, endodontic treatment is the best choice for preservation of teeth [2].

However, root canal treatment in primary teeth is technique sensitive, involving different steps that are complex and critical to the success of the procedures [3]. There are two major anatomical concerns that differentiate the treatment in teeth from that in permanent teeth. First, it is more difficult to establish the correct working length using radiographic methods due to the pattern of dental rhizolysis bevel [4, 5]. In addition, root canal preparation of this apical area must be extremely conservative, so as not to place the crown of the permanent successor tooth at risk. Therefore, laboratory research is warranted to investigate possible improvements in the technique of endodontic treatment in primary teeth, and to standardize the techniques used [6–8]. Nevertheless, for this type of research, there is an inherent difficulty in obtaining natural primary teeth with complete roots due to the physiological process of root resorption of these teeth.

Because of this fact, and to facilitate and improve teaching and laboratory training regarding endodontic treatment of primary teeth, artificial resin teeth have been developed to simulate the characteristics and structures of a natural tooth. These artificial teeth can also be used in endodontic research, replacing natural teeth. However, the feasibility of using these teeth for in vitro endodontic research and preclinical training needs to be evaluated.

Therefore, the present study aimed to evaluate the performance of artificial primary teeth for research purposes and the feasibility of their use, by comparing their results with results obtained with natural primary teeth, through an experiment investigating the accuracy of methods for determination of root canal length.

## Methods

### Description of artificial teeth

Artificial primary teeth (IM do Brasil Ltda., São Paulo, Brazil) were developed primarily with the objective of aiding undergraduate students in the training of endodontic treatment in primary teeth (patent pending with INPI/Brazil, register number PI 11001631–7).

**Fig. 1:**
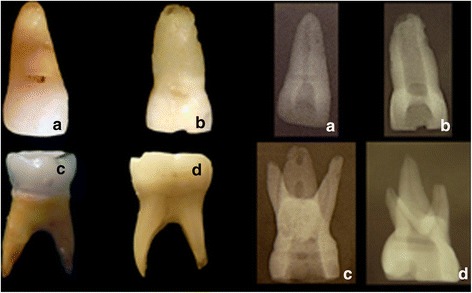
Visual and radiographic aspects of examples of artificial anterior (**a**) and posterior (**c**) primary teeth compared with natural anterior (**b**) and posterior (**d**) primary teeth

The artificial teeth are made with synthetic resin, comprising intact crown and roots that simulate upper primary incisors, canines, and upper and lower 2nd molars. Briefly, these teeth are manufactured using elastomer molds that are filled with a synthetic resin, forming two halves of the tooth. Then, the artificial pulp canals are filled with wax to simulate the pulp chamber and root canal content, and the parts are joined. They have external and internal anatomy similar to that of natural primary teeth, and present similar radiopacity, allowing the use of radiographic techniques (Fig. 1). Root resorption, however, is not simulated in the artificial teeth; they present entire roots.

### Ethical considerations and specimens preparation

This study was approved by the Local Ethics in Research Committee. Thirty natural primary incisors and 21 primary molars (78 root canals) were obtained, with root resorption of less than two-thirds of the total root length and no signs of internal or advanced external resorption in the furcation area. Those specimens were donated by the bank of teeth of University of São Paulo. The teeth were extracted as part of standard care, mainly for orthodontic reasons, and the children’s guardians donated them for the bank of teeth after signing a consent that they would be used for research purposes. The donation of the dental elements for our research was approved by the ethics in research committee. After the selection of the natural teeth, the same number of artificial teeth was selected (30 incisors and 21 primary molars with 63 root canals).

Preliminary periapical radiographs of each tooth were acquired to evaluate root canal anatomy and to verify the presence of morphological alterations. Then, the pulp chambers were accessed using a round bur in a high-speed handpiece, complemented with an Endo-Z bur (Dentsply Maillefer, Ballaigues, Switzerland). The root canals entrances were identified at the floor of the pulp chamber using an exploratory straight end probe.

Radicular pulp tissue or wax was removed by inserting and oscillating a No. #10 K-file (Dentsply Maillefer, Switzerland) to a point close to the apex. The canals were then irrigated with 1% sodium hypochlorite and dried using aspiration needles. No root canal preparation was performed prior to the determination of root canal length.

### Methods for root canal length determination

Three different methods were tested in the present study: a conventional radiographic method and two methods using two different electronic apex locators. For the radiographic method, a 70 kV, 8 mA X-ray device (Trophy Radiologie, Vincennes, France) with 0.4 s exposure time was used. Each tooth was fixed to an UltraSpeed D film (Eastman Kodak, Rochester, NY, USA) with sticky wax and then positioned perpendicular to the long axis of the tube, respecting a standardized distance of 15 cm. After film development in a dental film processor (A/T 2000; Air Techniques, Hicksville, NY, USA), root canal length was measured with a transparent plastic ruler (0.5 mm accuracy) at 2× magnification.

For the electronic methods, the measurements were carried out using two different electronic apex locator devices: Root ZX II (J. Morita Corp, Tokyo, Japan) and Mini Apex (SybronEndo, Orange, USA). The root portion of the teeth was embedded in floral foam impregnated with 0.9% sodium chloride solution [9] placed in individual plastic recipients (2.5 cm in diameter and 3.5 cm in height). The labial clip was also inserted in the foam prior to the assessment. The diameter of the endodontic file was chosen according to the canal size. Then, the root canal was moistened with 1% sodium hypochlorite and the selected K-file (Dentsply Maillefer, Baillaigues, Switzerland), attached to the file holder, was inserted into the root canal until the word ‘apex’ was visible on the display. The reading had to be stable for at least 5 s to determine the length. Once the reading was stable, the silicone stop was positioned and the root canal length was recorded using a 0.5-mm precision endodontic ruler.

Two operators conducted all methods twice with a 1-week interval between measurements.

### Reference standard method

For the reference standard method, a K-file (Dentsply Maillefer, Switzerland) with a silicone stop was inserted into the root canal until its tip was visible at either the apical foramen or the apical resorption bevel. Then, a different examiner made the assessments aided by a loupe (2× magnification). The silicone stop was placed at the coronal reference, and the file was laid against a metallic endodontic ruler of 0.5 mm precision to determine the root canal length.

### Statistical analysis

All analyses were performed separately for anterior and posterior primary teeth. Inter- and intraexaminer agreement was evaluated using the intraclass correlation coefficient (ICC) and respective 95% confidence intervals (95%CI). The second assessment made by each examiner was used to calculate the intraexaminer reliability. For the subsequent analysis, we only considered the first evaluations of one examiner, since both examiners reached similar findings in their assessments.

To evaluate the validity of the methods, the results obtained with each method were compared with the actual root canal length obtained using the reference standard. First, the validity was calculated through Bland-Altman analysis, which permitted evaluation of systematic differences and the 95% limits of agreement between the values obtained with each method and the actual root canal length.

Absolute differences between the root canal length obtained with the three methods and that obtained with the reference standard were calculated. We carried out the Anderson-Darling test to check the normality, and Levene test to evaluate the homogeneity of variances of these values. As the differences between the values obtained using the reference standard method and those obtained using the three methods were not normally and homogeneously distributed, they were compared using the Friedman test.

All analyses were performed using appropriate statistical software (MedCalc 12.1.4.0, MedCalc Software bvba, Ostend, Belgium). The level of significance was set at *p* < 0.05.

## Results

Regarding the intra-examiner reproducibility, we observed that all methods presented high ICC values in anterior and posterior using both natural or artificial primary teeth, with values higher than 0.78 for all methods. For interexaminer reliability, ICC values were slightly lower; however, all methods achieved values higher than 0.7. We considered that all methods reached a good consistency for both groups of teeth (Tables 1 and 2). However, regarding the inter-examiner reliability, there was a trend of lower values obtained in artificial anterior teeth, mainly for Root ZX (Table 1).

**Table 1:** Inter- and intra-examiner reproducibility for natural and artificial anterior teeth among the methods used for canal length determination through Intraclass Correlation Coefficient calculation

Methods for canal length determination	Intraexaminer reliability		Interexaminer reliability	
	Examiner 1	Examiner 2	1st evaluation	2nd evaluation
	ICC (95% CI)	ICC (95% CI)	ICC (95% CI)	ICC (95% CI)
Natural anterior teeth (*n* = 30)				
Radiographic	0.983 (0.966 to 0.992)	0.993 (0.986 to 0.997)	0.921 (0.839 to 0.962)	0.923 (0.832 to 0.964)
Mini Apex	0.974 (0.945 to 0.987)	0.992 (0.983 to 0.996)	0.909 (0.818 to 0.956)	0.943 (0.884 to 0.972)
Root ZX	0.994 (0.987 to 0.997)	0.988 (0.973 to 0.995)	0.959 (0.916 to 0.980)	0.959 (0.916 to 0.980)
Artificial anterior teeth (*n* = 30)				
Radiographic	0.982 (0.958 to 0.992)	0.968 (0.934 to 0.985)	0.939 (0.729 to 0.979)	0.960 (0.838 to 0.985)
Mini Apex	0.789 (0.603 to 0.894)	0.949 (0.895 to 0.975)	0.870 (0.746 to 0.935)	0.809 (0.637 to 0.905)
Root ZX	0.895 (0.791 to 0.949)	0.947 (0.891 to 0.974)	0.760 (0.557 to 0.878)	0.709 (0.475 to 0.850)

*ICC* Intraclass correlation coefficients, *95% CI* 95% confidence intervals

**Table 2:** Inter- and intra-examiner reproducibility for natural and artificial posterior teeth among the methods used for canal length determination through Intraclass Correlation Coefficient calculation

Methods for root length determination	Intraexaminer reliability		Interexaminer reliability	
	Examiner 1	Examiner 2	1st evaluation	2nd evaluation
	ICC (95% CI)	ICC (95% CI)	ICC (95% CI)	ICC (95% CI)
Natural posterior teeth (*n* = 78 canals in 21 teeth)				
Radiographic	0.980 (0.968 to 0.987)	0.966 (0.947 to 0.978)	0.978 (0.956 to 0.988)	0.982 (0.970 to 0.989)
Mini Apex	0.984 (0.976 to 0.990)	0.995 (0.991 to 0.997)	0.933 (0.896 to 0.957)	0.936 (0.902 to 0.959)
Root ZX	0.986 (0.979 to 0.991)	0.992 (0.986 to 0.995)	0.949 (0.921 to 0.967)	0.933 (0.897 to 0.957)
Artificial posterior teeth (*n* = 63 canals in 21 teeth)				
Radiographic	0.976 (0.960 to 0.985)	0.983 (0.970 to 0.990)	0.912 (0.858 to 0.946)	0.929 (0.885 to 0.956)
Mini Apex	0.988 (0.981 to 0.993)	0.994 (0.991 to 0.997)	0.971 (0.952 to 0.982)	0.963 (0.939 to 0.977)
Root ZX	0.993 (0.988 to 0.996)	0.997 (0.994 to 0.998)	0.959 (0.933 to 0.975)	0.959 (0.932 to 0.976)

***ICC*** Intraclass correlation coefficients, ***95% CI*** 95% confidence intervals

Considering the anterior teeth, with the Bland-Altman analysis, we observed that the use of the electronic apex locators devices resulted in a better correspondence with the actual root canal length obtained by the reference standard method for both natural and artificial teeth, since the range between the 95% limits of agreement was narrower than those for the other methods (Table 3). This trend was similar between the groups of teeth, although the radiographic method presented wider ranges, indicating inferior accuracy values compared with the electronic apex locators. The differences between the actual length and the measures obtained by the methods showed values lower than 1.0 mm with all methods. Considering ICC values, however, the performance of both electronic apex locators used in the present study was significantly lower in the artificial teeth than in the natural anterior primary teeth (Table 3).

**Table 3:** Accuracy of the methods used for canal length determination through Intraclass Correlation Coefficient calculation and Bland-Altman analysis for natural and artificial anterior teeth

Methods for canal length determination	ICC (95% CI)	Bland-Altman analysis		
		Mean difference	Upper limit	Lower limit
Natural anterior teeth (*n* = 30)				
Radiographic	0.882 (0.695 to 0.949)	−0.52	1.20	−2.23
Mini Apex	0.962 (0.914 to 0.983)	0.22	1.20	−0.77
Root ZX	0.945 (0.749 to 0.981)	0.42	1.42	−0.58
Artificial anterior teeth (*n* = 30)				
Radiographic	0.518 (−0.038 to 0.792)	−0.87	0.72	−2.45
Mini Apex	0.644 (0.031 to 0.863)	0.66	1.90	−0.58
Root ZX	0.617 (0.267 to 0.810)	0.43	1.86	−0.99

***ICC*** Intraclass correlation coefficients, *95% CI* 95% confidence intervals

Regarding the posterior primary molars, the same trends were observed for both types of teeth (Table 4). The electronic apex locators presented superior accuracy than the radiographic method. The differences between the actual length values and the values obtained through all methods were lower than 2.0 mm for all methods, but this difference was very small for the electronic apex locator devices (up to 0.4 mm). The differences between the results in artificial and natural teeth were more pronounced for the radiographic method.

**Table 4:** Accuracy of the methods used for canal length determination through Intraclass Correlation Coefficient calculation and Bland-Altman analysis for natural and artificial posterior teeth

Methods for canal length determination	ICC (95% CI)	Bland-Altman analysis		
		Mean difference	Upper limit	Lower limit
Natural posterior teeth (*n* = 78 canals in 21 teeth)				
Radiographic	0.685 (0.545 to 0.787)	−0.40	2.90	−3.70
Mini Apex	0.941 (0.907 to 0.962)	0.18	1.54	−1.19
Root ZX	0.958 (0.935 to 0.973)	0.14	1.33	−1.05
Artificial posterior teeth (*n* = 63 canals in 21 teeth)				
Radiographic	0.625 (−0.037 to 0.853)	−1.70	1.00	−4.40
Mini Apex	0.959 (0.914 to 0.978)	0.29	1.45	−0.88
Root ZX	0.947 (0.855 to 0.975)	0.40	1.64	−0.85

***ICC*** Intraclass correlation coefficients, *95% CI* 95% confidence intervals

The only method that showed an overestimation of canal lengths (values higher than actual lengths) for both anterior and posterior primary teeth was the radiographic method. This result was observed for both natural and artificial teeth (Tables 3 and 4). The other methods presented lower values than the measure obtained with the reference standard. Considering the ICC values, it was observed that the measurements made in the artificial anterior and posterior teeth using the radiographic examination presented great variability, since the 95% CI values were very large (Tables 3 and 4).

**Fig. 2:**
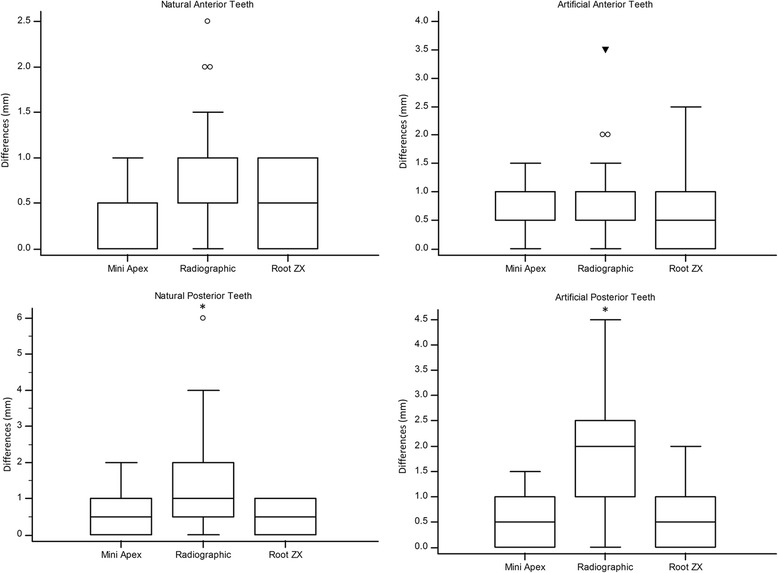
Box-and-Whisker plots of absolute difference between the actual length and those obtained by the methods of root canal length determination (mm). Asterisks represent that the method presented statistical significant difference (*p* < 0.05 though Friedman test) from other methods considering the same group of teeth

Considering the absolute differences from the actual root canal length, in both artificial and natural anterior teeth, the median of differences obtained with all methods were up to 1.0 mm, and there were no differences among the methods. On the other hand, for posterior teeth, the radiographic method presented significant greater differences compared with the electronic apex locator methods, for both natural and artificial teeth (Fig. 2).

## Discussion

In contrast to endodontic treatment in permanent teeth, root canal treatment in primary teeth presents some problems that must be addressed in order to optimize treatment success rates [6–8]. This optimization can be developed through laboratory research, which aims to improve the quality of the different treatment steps. However, since extracted or exfoliated primary teeth usually present a great extension of root resorption due to physiological or pathological processes, sample collection for this type of research is laborious. The same difficulty occurs in collecting teeth for training and educational purposes.

For those reasons, development of artificial primary teeth would be useful. The artificial teeth used in the present study show an acceptable similarity with natural ones, and have been used for training and teaching of undergraduate and graduate students in our dental school. The teeth present similar external and internal anatomy, and also exhibit radiopacity comparable to natural primary teeth. Nevertheless, the effectiveness of this procedure in improving students’ skills in the endodontic treatment of primary teeth has not been tested yet.

A recently published study [10] with permanent teeth compared preclinical training in natural and artificial teeth regarding students’ performance. The authors observed the training using artificial permanent teeth was suitable in preparing students for endodontic treatment. Similar studies with artificial primary teeth should be also performed.

However, the interest of the present investigation was to test artificial teeth for research purposes, more specifically, in an experiment for canal length determination. It is still unclear if this similarity would be adequate also for research purposes. To the best of our knowledge, no previous studies have tested the utilization of artificial primary teeth as substitutes for natural primary teeth.

With regard to the performance of the methods for canal length determination tested in the present study, earlier studies confirmed better accuracy of electronic apex locators for determining root canal length compared with other methods [4, 11–13]. The findings of this study corroborate the better performance found in previous studies for the two different brands of these devices. Because the accuracy of electronic apex locators is influenced by moisture content in root canals and the diameter of the apical foramen, these devices were initially thought to be useless for primary teeth. Some studies reported the critical diameter of the apical foramen for accurate measurements ranges from 0.20 to 0.62 mm [14, 15]. In addition, many studies concluded that electronic apex locators do not give precise results in permanent teeth with open apices [14–16]. However, in more recent studies evaluating whether root resorption of primary molars affects the precision of electronic apex locators, [17, 18], it was demonstrated that the accuracy of the device is good even when this physiologic event is present.

Still, the most used technique in daily clinical practice is the radiographic method. This method, however, was the least precise technique evaluated. These results might be related to the way the measurements are made using the imaging methods. The trend in overestimating the actual canal length observed with this method could be due to difficulty in determining the correct position of the apex, mainly because of the physiological resorption process. For imaging methods, root length determination is obtained linearly, whereas for electronic methods, a flexible file is inserted into the root canal. Since the file can curve according the shape of the root canal, the measure obtained can approximate the actual length much more accurately. This fact might also explain why the imaging methods usually performed better for anterior teeth in our study.

Despite the worst performance obtained with the radiographic method in the present study, the reproducibility and accuracy obtained with this method were still good. Radiography allows clinicians accessible, cost-effective, and relatively good image resolution; it continues to be of value in endodontic therapy. Moreover, a systematic review on the accuracy of electronic apex locators compared to radiographic methods, to determine root canal length of permanent teeth, show that clinically significant differences between the methods are small [13]. Thus, radiographies are still useful for correct diagnosis and treatment planning regarding endodontic therapy of primary teeth. In daily clinical practice, used to determine the working canal length. However, caution should be taken to avoid repetition of radiographies due to the hazards of ionizing radiation. Moreover, further clinical studies should be conducted to investigate the performance of the radiographic method in determining root canal length under more realistic conditions.

Considering the main objective of the study, the comparison between results obtained with artificial and natural primary teeth, in general, similar tendencies for both types of teeth were observed. Nevertheless, some points should be noted. For anterior primary teeth, for example, the precision obtained with all methods was lower in the assessments made in the artificial sample. Even with the electronic methods, lower values of reliability were obtained in the artificial anterior teeth.

Another possible explanation for the differences observed could be related to the differences in the apical region of the teeth. As previously mentioned, artificial teeth present entire roots, with no simulation of physiological root resorption that occurs in natural primary teeth. Although previous studies reported that root resorption exerts little influence on the accuracy of the methods for canal length determination [4, 17, 18], these differences could explain the differences observed in the present study.

Furthermore, the variability of measurements made with the radiographic method was more noticeable in the artificial teeth, since a wide extension of confidence intervals was observed in both anterior and posterior teeth. Although artificial teeth have the same root length, they present many internal anatomical differences, probably due to the variability in the manufacturing process. Another possible explanation for the inconsistency in results of the radiographic method could be differences related to the radiopacity of the artificial teeth.

Previous studies tested another potential method for laboratory research, namely simulated canals [19, 20]. Nevertheless, this model does not recreate the complex anatomy of pulp chambers and root canals. Artificial teeth, such as those used in the present study, could be a better alternative for educational and research purposes, since they are purchased ready for use. However, due to the findings obtained in the present study, although this model has good potential, some improvements are needed, at least for research purpose regarding experiments of canal length determination. Moreover, further studies to investigate other steps of endodontic treatment of primary teeth, such as instrumentation and canal filling, should be conducted using artificial teeth, in order to evaluate the feasibility of their use.

## Conclusions

Artificial primary teeth are similar to natural primary teeth, and experiments on canal length determination could be carried out using this model. However, because of the variability of the results obtained, and some differences observed, the extrapolation of the findings of the present study is limited; hence, it is possible to conclude that artificial primary teeth will need improvements in order to be employed as substitutes for natural primary teeth in endodontic laboratory research.

## Additional files

Additional file 1: Data used in research analysis. Numerical values of the data used in the analysis, separated by type of teeth in 4 different sheets: Natural anterior, Artificial anterior, Natural posterior, and Artificial posterior. (XLSX 22 kb)

## Abbreviations

95% CI: 95% confidence interval
ICC: Intraclass correlation coefficient

## References

[1] Kopel HM (1970). Root canal therapy for primary teeth. J Mich State Dent Assoc.

[2] American Academy of Pediatric Dentistry (2008). Guideline on pulp terapy for primary and young permanent teeth. Pediatr Dent.

[3] Journal of Endodontics (2008). Editorial board: success and failure in Endodontics: an online study guide. J Endod.

[4] Mello-Moura AC, Moura-Netto C, Araki AT, Guedes-Pinto AC, Mendes FM (2010). Ex vivo performance of five methods for root canal length determination in primary anterior teeth. Int Endod J.

[5] Leonardo MR, Silva LA, Nelson-Filho P, Silva RA, Raffaini MS (2008). Ex vivo evaluation of the accuracy of two electronic apex locators during root canal length determination in primary teeth. Int Endod J.

[6] Nadin G, Goel BR, Yeung CA, Glenny AM. Pulp treatment for extensive decay in primary teeth. Cochrane Database Syst Rev. 2003;1:CD003220. 10.1002/14651858.CD003220 12535462

[7] Smail-Faugeron V, Courson F, Durieux P, Muller-Bolla M, Glenny AM, Fron Chabouis H (2014). Pulp treatment for extensive decay in primary teeth. Cochrane Database Syst Rev.

[8] Mejare IA, Klingberg G, Mowafi FK, Stecksen-Blicks C, Twetman SH, Tranaeus SH (2015). A systematic map of systematic reviews in pediatric dentistry--what do we really know?. PLoS One.

[9] Guerreiro-Tanomaru JM, Croti HR, Silva GF, Tanomaru-Filho M (2012). Tooth embedding medium influences the accuracy of electronic apex locator. Acta Odontol Latinoam.

[10] Bitter K, Gruner D, Wolf O, Schwendicke F (2016). Artificial versus natural teeth for preclinical endodontic training: a randomized controlled trial. J Endod.

[11] Ahmad IA, Pani SC (2015). Accuracy of electronic apex locators in primary teeth: a meta-analysis. Int Endod J.

[12] Kielbassa AM, Muller U, Munz I, Monting JS (2003). Clinical evaluation of the measuring accuracy of ROOT ZX in primary teeth. Oral Surg Oral Med Oral Pathol Oral Radiol Endod.

[13] Martins JN, Marques D, Mata A, Carames J (2014). Clinical efficacy of electronic apex locators: systematic review. J Endod.

[14] Fouad AF, Rivera EM, Krell KV (1993). Accuracy of the Endex with variations in canal irrigants and foramen size. J Endod.

[15] Saito T, Yamashita Y (1990). Electronic determination of root canal length by newly developed measuring device. Influences of the diameter of apical foramen, the size of K-file and the root canal irrigants. Dent Jpn (Tokyo).

[16] Kim YJ, Chandler NP (2013). Determination of working length for teeth with wide or immature apices: a review. Int Endod J.

[17] Angwaravong O, Panitvisai P (2009). Accuracy of an electronic apex locator in primary teeth with root resorption. Int Endod J.

[18] Nelson-Filho P, Lucisano MP, Leonardo MR, da Silva RA, da Silva LA (2010). Electronic working length determination in primary teeth by ProPex and digital signal processing. Aust Endod J.

[19] Dummer PM, Alodeh MH, Al-Omari MA (1991). A method for the construction of simulated root canals in clear resin blocks. Int Endod J.

[20] Petersson K, Olsson H, Soderstrom C, Fouilloux I, Jegat N, Levy G (2002). Undergraduate education in endodontology at two European dental schools. A comparison between the Faculty of Odontology, Malmo university, Malmo, Sweden and Faculty of Odontology, Paris 5 university (Rene Descartes), France. Eur J Dent Educ.

